# Individual pain sensitivity is associated with resting-state cortical activities in healthy individuals but not in patients with migraine: a magnetoencephalography study

**DOI:** 10.1186/s10194-020-01200-8

**Published:** 2020-11-16

**Authors:** Fu-Jung Hsiao, Wei-Ta Chen, Hung-Yu Liu, Yen-Feng Wang, Shih-Pin Chen, Kuan-Lin Lai, Li-Ling Hope Pan, Shuu-Jiun Wang

**Affiliations:** 1 Brain Research Center, National Yang-Ming University, Taipei, Taiwan; 2 School of Medicine, National Yang-Ming University, Taipei, Taiwan; 3 Department of Neurology, Neurological Institute, Taipei Veterans General Hospital, Taipei, Taiwan

**Keywords:** Pain sensitivity, Resting state, Oscillation, Gamma, Functional connectivity, Episodic migraine, Magnetoencephalography

## Abstract

**Background:**

Pain sensitivity may determine the risk, severity, prognosis, and efficacy of treatment of clinical pain. Magnetic resonance imaging studies have linked thermal pain sensitivity to changes in brain structure. However, the neural correlates of mechanical pain sensitivity remain to be clarified through investigation of direct neural activities on the resting-state cortical oscillation and synchrony.

**Methods:**

We recorded the resting-state magnetoencephalographic (MEG) activities of 27 healthy individuals and 30 patients with episodic migraine (EM) and analyzed the source-based oscillatory powers and functional connectivity at 2 to 59 Hz in pain-related cortical regions, which are the bilateral anterior cingulate cortex (ACC), medial orbitofrontal (MOF) cortex, lateral orbitofrontal (LOF) cortex, insula cortex, primary somatosensory cortex (SI), primary motor cortex (MI), and posterior cingulate cortex (PCC). The mechanical punctate pain threshold (MPPT) was obtained at the supraorbital area (the first branch of the trigeminal nerve dermatome, V1) and the forearm (the first thoracic nerve dermatome, T1) and further correlated with MEG measures.

**Results:**

The MPPT is inversely correlated with the resting-state relative powers of gamma oscillation in healthy individuals (all corrected *P* < 0.05). Specifically, inverse correlation was noted between the MPPT at V1 and gamma powers in the bilateral insula (r = − 0.592 [left] and − 0.529 [right]), PCC (r = − 0.619 and − 0.541) and MI (r = − 0.497 and − 0.549) and between the MPPT at T1 and powers in the left PCC (*r* = − 0.561) and bilateral MI (*r* = − 0.509 and − 0.520). Furthermore, resting-state functional connectivity at the delta to beta bands, especially between frontal (MOF, ACC, LOF, and MI), parietal (PCC), and sensorimotor (bilateral SI and MI) regions, showed a positive correlation with the MPPT at V1 and T1 (all corrected *P* < 0.05). By contrast, in patients with EM, the MPPT was not associated with resting-state cortical activities.

**Conclusions:**

Pain sensitivity in healthy individuals is associated with the resting-state gamma oscillation and functional connectivity in pain-related cortical regions. Further studies must be conducted in a large population to confirm whether resting-state cortical activities can be an objective measurement of pain sensitivity in individuals without clinical pain.

**Supplementary Information:**

The online version contains supplementary material available at 10.1186/s10194-020-01200-8.

## Introduction

Pain sensitivity may determine the risk, severity, prognosis, and treatment efficacy of clinical pain [1, 2]. Reduced pain sensitivity may delay recognition and undermine treatment efficacy in acute pain [3], whereas elevated pain sensitivity may increase health care costs and susceptibility to chronic pain conditions [4]. In the era of precision medicine, objective assessment of pain sensitivity at the individual level is an unmet need. Pain sensitivity is highly variable across individuals. Notably, genetic [5, 6], environmental [7, 8], and psychological [9, 10] factors influence individual pain perception [11].

Some studies have explored the neural correlates of pain sensitivity. Studies involving structural magnetic resonance imaging (MRI) have identified that cortical thickness or volume density change in the pain-related cortical network may contribute to varying pain sensitivity across healthy individuals. More specifically, high pain sensitivity was associated with cortical thickening in the primary somatosensory cortex (SI), posterior cingulate cortex (PCC), and orbitofrontal cortex [12]. Additionally, the intensity rating of thermal pain was negatively correlated with grey matter density in the SI, PCC, precuneus, intraparietal sulcus, and inferior parietal cortex [13]. In line with the aforementioned MRI studies, neurophysiological studies obtaining direct neural signals showed that noxious stimuli activated a widely distributed brain network related to pain processing, including the SI, primary motor cortex (MI), insula, anterior cingulate cortex (ACC), medial frontal cortex, and PCC [14]. Moreover, activations among some of these regions were greater in pain-sensitive individuals than in pain-insensitive ones [15], and the magnitude increased with increasing stimulus intensity or perceived magnitude of pain intensity [16]. The aforementioned findings suggest the pain-related cortical network as the structural correlate of individual thermal pain sensitivity, and noxious-evoked neural oscillations and synchronizations in these regions may cause the intersubject variability in pain perception. However, how the neural correlates of spontaneous cortical activities with the mechanical pain sensitivity and in clinical pain remains elusive.

The rating of individual pain in response to noxious stimulus has been coded from cortical activation [16–19], and pain intensity during heat stimulation was related to gamma oscillation in the medial frontal cortex [19]. In another study, during capsaicin-heat pain, peak alpha frequency over the sensorimotor region was inversely correlated with individual pain intensity [18]. The inconsistent findings regarding cortical regions and oscillations may be attributable to the influence of the pain modalities and the involvement of cognitive processes, such as the salience or attention effect. Therefore, some studies have investigated prestimulus functional connectivity instead of stimulus-evoked responses and found that the connectivity of the anterior insula cortex [20] and frontocentral network [21] determined the pain perception of the subsequent noxious stimulus. Thus, brain activities or synchrony might involve the neurophysiological mechanisms for individual pain perception. Moreover, brain oscillations and synchrony serve integrative functions through flexibly regulating information flow among the cortical regions [22–24]. Thus, exploring oscillation and synchrony during the resting-state condition might yield promising insights into how functionally diverse processes relevantly reflect the intersubject variability of pain sensitivity.

This study investigates the hypothesis that resting-state cortical activities at the pain-related cortical network underpin interindividual pain variability. To characterize temporal–spatial features of cortical oscillations and cortico-cortical synchronization within this pain-related network, the present study used magnetoencephalography (MEG) to record brain activities during the resting-state condition. Moreover, this study assessed individual pain sensitivity with the mechanical punctate pain threshold (MPPT) instead of the thermal pain threshold used in most of the earlier pertinent studies. Furthermore, we recruited patients with episodic migraine (EM) to determine whether the study findings are exclusive to patients with pain disorder. We selected EM here because patients with EM have been characterized as having an aberrant pain sensitivity threshold [25], heightened cortical excitability [26, 27], and altered resting-state cortical oscillations and connectivity in pain-related regions [28–30]. Moreover, whether the underlying pain sensitivity mechanism is reshaped for pain disorder remains uncertain. The specific aims of this study were to (1) elucidate the relationship between cortical oscillations and pain sensitivity, (2) investigate the correlation of cortical synchronization with pain sensitivity, and (3) examine the effect of pain disorder in patients with EM on the cortical mechanism of pain sensitivity.

## Methods

### Participants

This study recruited healthy individuals who did not have medical or family histories of pain disorders and had not experienced any significant pain condition during the past year. Furthermore, patients with EM (monthly headache days: 1–14) were enrolled from the Headache Clinic of Taipei Veterans General Hospital (VGHTPE), and the diagnosis (code 1.1: migraine without aura) was according to the criteria of the *International Classification of Headache Disorders, Third Edition* [31]. All participants were right-handed, denied having any history of systemic or major neuropsychiatric disease, and had normal physical and neurological examination results as well as normal brain MRI results. Participants who were taking any medication (eg, migraine preventive medications) on a daily basis were excluded. Furthermore, patients with migraine who overused medication were excluded. The hospital’s institutional review board (IRB) approved the study protocol (VGHTPE-IRB, 2015–10-001 BC), and each participant provided written informed consent.

All participants underwent scheduled MPPT and MEG recordings (both detailed below) and were instructed not to take any analgesics or other medications within 3 days before the recordings. Patients with migraine were assessed during their interictal phase, which was defined as the absence of acute migraine within 2 days before (days − 1 and − 2) and after (days + 1 and + 2) the aforementioned assessment. We evaluated the severity of depression and anxiety using the Hospital Anxiety and Depression Scale (HADS) and functional disability in migraine using the Migraine Disability Assessment Scale (MIDAS).

### Pain sensitivity measurement

Pain sensitivity measurement was performed in the chronic pain examination room (constant 20 °C room temperature and no windows) in the Department of Neurology at VGHTPE. Quantitative measurements of the MPPT were defined as the lowest intensity perceived as painful for participants, and MPPT values were obtained as follows. MPTT was determined using the standard rigid electronic von Frey device (ALMEMO 2450, AHLBORN, Germany), which consists of a 1000-g internal load cell connected to a probe and a rigid tip (diameter: 0.8 mm; IITC Life Science Inc., USA). The system digitally measures and displays the values in grams, with a resolution of 0.1 g. During measurement, the tip was applied perpendicularly to the skin surface, with ascending stimulus intensity applied at 25 g/s [32]. Participants were instructed to inform the examiner immediately upon experiencing a painful sensation. Assessment target areas included the left supraorbital (ie, the first branch of the trigeminal nerve dermatome, V1) and proximal medio-ventral forearm (ie, the first thoracic nerve dermatome, T1). Breaks were taken between each stimulus, and the order of stimulation sites was randomized. Sensory stimuli on each target area were delivered 5 times; the 2 extreme values were excluded, and the average values of the remaining 3 recordings were calculated for further analyses.

### Resting-state MEG recording

A 5-min resting-state MEG recording [33, 34] was obtained for each participant, each of whom sat comfortably with eyes closed but remained awake and relaxed. If the participant fell asleep or had excessive within-run head movement, the recording was stopped and then rerun. Furthermore, a 3-min empty-room recording was conducted to capture sensor and environmental noises, which were applied to calculate the noise covariance for further source model analysis. MEG data were recorded with the digitization rate of 600 Hz using a whole-scalp 306-channel neuromagnetometer (Vectorview; Elekta Neuromag, Helsinki, Finland) composed of 102 identical triple sensor elements (1 magnetometer and 2 orthogonal planar gradiometers). In total, 4 coils representing the head position were placed on the participant’s scalp, specified by the nasion and 2 preauricular points using Cartesian coordinates and measured with a 3-dimensional (3D) digitizer. For accurate registration, approximately 50 additional scalp points were digitized. These head landmarks and points enabled further coordinate alignment between the MEG and MRI data. Additionally, electrooculography (EOG) and electrocardiography (ECG) activities during MEG recording were simultaneously recorded for data preprocessing. MRI images of individual brain structures were acquired using a 3 T MR system (Siemens Magnetom Tim Trio), with a TR of 9.4 ms, TE of 4 ms, recording matrix of 256 × 256 pixels, field of view of 256 mm, and slice thickness of 1 mm.

### Data preprocessing

**Fig. 1:**
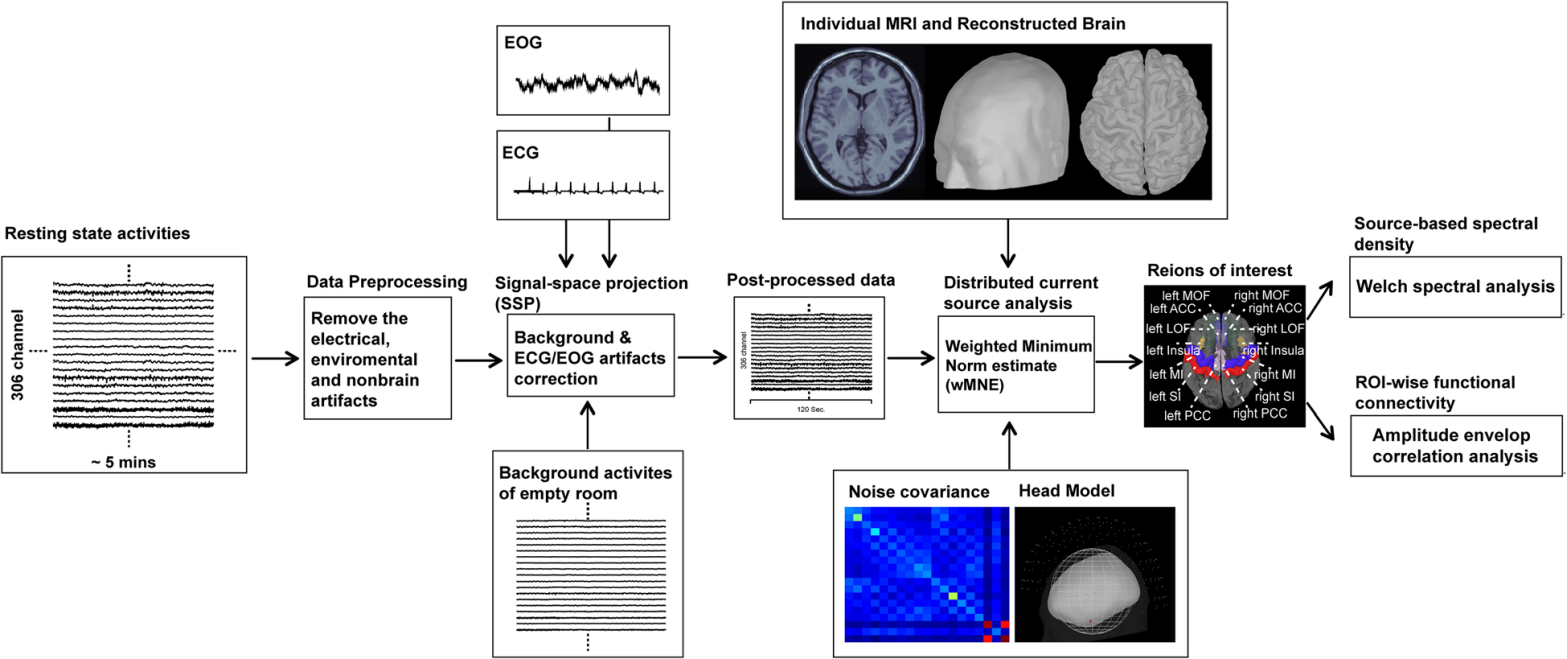
Pipeline of source-based resting-state magnetoencephalographic spectral and functional connectivity analysis

To obtain intrinsic spontaneous cortical activities and reject the contaminations of nonbrain or environmental artifacts, MEG data were preprocessed as follows (Fig. 1): (1) MaxFilter from the Neuromag software system was applied to remove external noise from MEG recordings [35, 36], (2) the data segments containing artifacts from head movement, muscle activities or environmental noise were manually rejected, (3) notch filters (60 Hz and its harmonics) were used to remove powerline contaminations, (4) identified heartbeat and eye blinking events from ECG and EOG data were used to define the projectors through principal component analysis separately. The principal components meeting the artifact’s sensor topography were then manually excluded through orthogonal projection [37].

Furthermore, for further source modeling analysis, T1-weighted structural volumetric images were automatically reconstructed into a surface model using BrainVISA (4.5.0, http://brainvisa.info). The detailed geometric reconstruction of the scalp, brain gray and white matter, and tessellations provided a topographical 3D representation of the brain surface and was used to estimate the gray and white matter border.

### Source-based spectral power and functional connectivity analysis

In combination with a structural surface model, the distributed source model of resting-state MEG data was estimated using depth-weighted minimum norm estimation analysis (Fig. 1). A forward model was established by overlapping sphere method for rapid calculation of realistic head shapes [38], which presented each cortical vertex as a current dipole and included approximately 15,000 vertices in the whole brain model. The inverse operator estimated the distribution of current sources that account for data recorded at the MEG sensors. For group analysis, the cortical source model of each participant was then morphed into a common source space defined by the Colin27 anatomy [39]. Through dynamic source analysis, the current density of cortical activation in each individual could be obtained at any cortical region and any time point.

In this study, the regions of interest (ROIs) were defined in the T1 template volume using Mindboggle cortical parcellation [40] and were composed of 14 brain regions, including the bilateral ACC, medial orbitofrontal cortex (MOF), lateral orbitofrontal cortex (LOF), insula cortex, SI, MI, and PCC. These ROIs were involved in the sensory and affective aspects of cortical pain representation and were related to pain sensitivity in terms of the structural volume or thickness [12–14]. Besides, the bilateral auditory cortices, apart from the pain regions, were also selected as negative control. The time-varying source density of each ROI was individually derived from the averaged source density of each vertex within the ROI. To obtain the resting-state oscillations and functional connectivity, the dynamic source density of each ROI was further analyzed as follows. First, oscillatory power at each ROI was estimated using the Welch method (window duration: 5 s with 50% overlap) and defined as absolute power. Moreover, the oscillatory power was normalized to relative power through the division of the power at each frequency band by the total power, which has been reported to adequately reduce the interindividual variability of the oscillatory magnitude [41]. Second, the amplitude envelope correlation analysis [42], which orthogonalized the signals to remove zero-lag interactions [43], was used to calculated the oscillatory functional connectivity between ROIs and then constructed the full 14 × 14 adjacency matrix. Oscillatory power and functional connectivity were categorized according to frequency bands: delta (2–4 Hz), theta (5–7 Hz), alpha (8–13 Hz), beta (14–29 Hz), and gamma (30–59 Hz), and averaged in each frequency range. The MEG data preprocessing and analysis were performed using Brainstorm [44].

### Statistical analysis

The demographics and MPPT of the healthy individuals and EM were compared using Student’s *t* test or a chi-square test as appropriate. Pearson’s correlation was used to determine the correlation between the MPPT (V1 and T1) and cortical oscillations (absolute and relative) and synchronization (14 × 14 ROIs) at the delta to gamma frequency bands, as well as the oscillations and synchronization in the bilateral auditory cortex. Throughout the statistical analyses, false discovery rate correction (FDR) was used for multiple comparisons, and a *P* value of < 0.05 was considered statistically significant.

## Results

### Demographics and pain sensitivity responses

This study evaluated 57 participants (27 healthy individuals and 30 patients with EM). The groups did not differ in age and sex (both *P* > 0.05; Table 1). The MPPT at V1 was higher in patients with EM than in healthy individuals (*P* = 0.041); however, no difference was observed at T1. Moreover, the anxiety and depression scores were higher in patients with EM than in healthy individuals (all *P* < 0.01).

**Table 1:** Subject Characteristics

		Healthy control (*N* = 27)	Episodic migraine (*N* = 30)
Age		39.9 ± 9.8	37.0 ± 10.2
Sex		6 M/21F	5 M/25F
MPPT (g)	V1	85.3 ± 33.5	103.5 ± 32.2*
	T1	87.3 ± 42.8	80.3 ± 26.8
HADS-A		3.4 ± 2.5	6.8 ± 3.6*
HADS-D		3.3 ± 2.9	8.0 ± 5.9*
Headache frequency (/month)		–	6.0 ± 2.9
MIDAS		–	27.0 ± 28.5

*MPPT* Mechanical punctate pain threshold, *V1* Left supraorbital (the first branch of trigeminal nerve dermatome), *T1* Proximal medio-ventral forearm (the first thoracic nerve dermatome), *HADS-A* The anxiety subscale of the Hospital Anxiety and Depression Scale, *HADS-D* The depression subscale of the Hospital Anxiety and Depression Scale, *MIDAS* Migraine Disability Assessment. *, *p* < 0.05

### Correlations between resting-state cortical oscillations and the MPPT

**Fig. 2:**
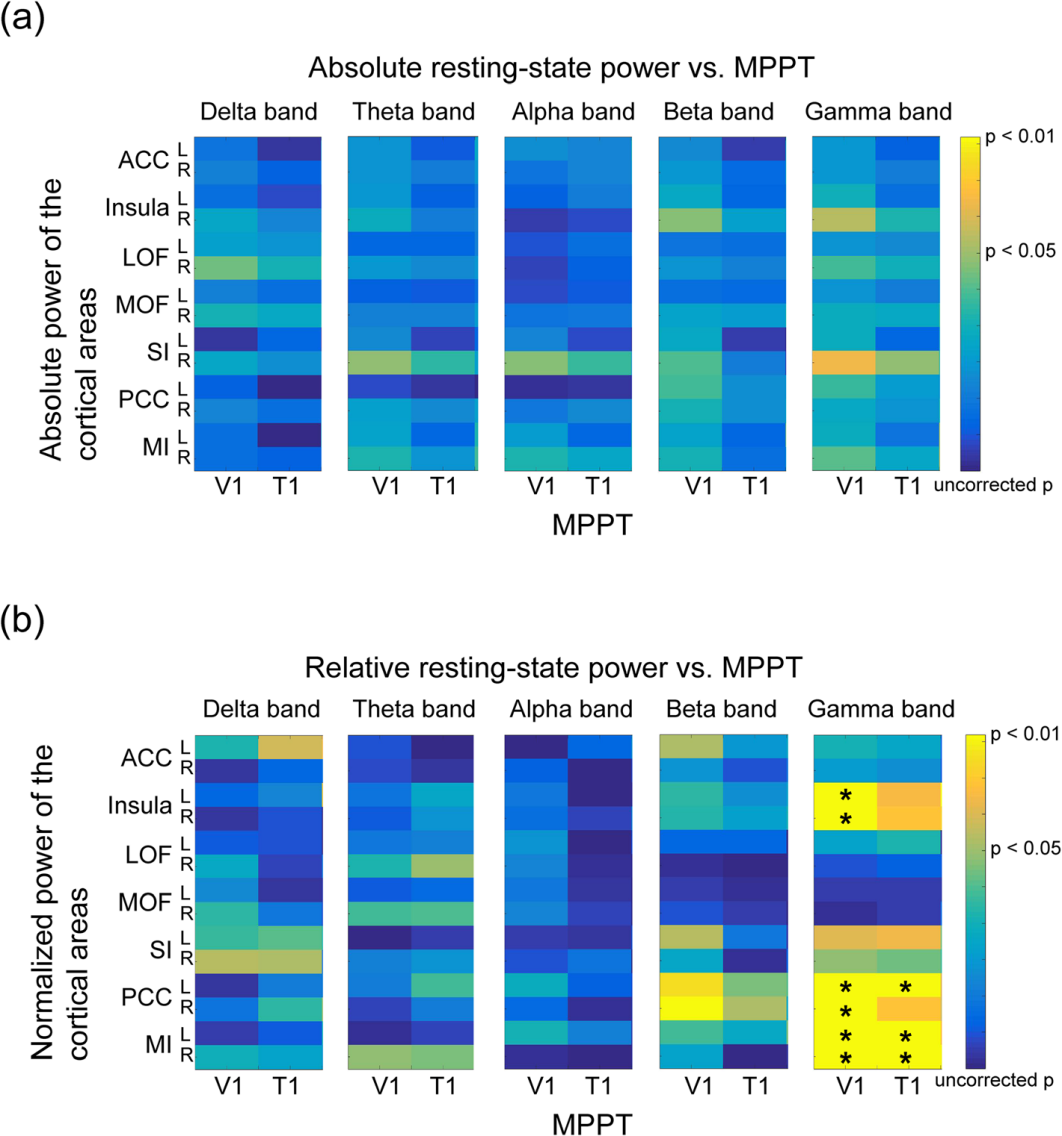
Relationship of the mechanical punctate pain threshold with the (**a**) absolute power and (**b**) relative power at the delta to gamma bands in the pain-related regions in healthy individuals. The *P*-value of the correlation analysis is color-coded, with a significant value denoted with yellow. *P < .05 with false discovery rate correction. MPPT, mechanical punctate pain threshold; ACC, anterior cingulate cortex; LOF, lateral orbitofrontal cortex; MOF, medial orbitofrontal cortex; SI, primary somatosensory cortex; PCC, posterior cingulate cortex; MI, primary motor cortex; V1, left supraorbital (the first branch of the trigeminal nerve dermatome); T1, proximal medio-ventral forearm (the first thoracic nerve dermatome)

**Fig. 3:**
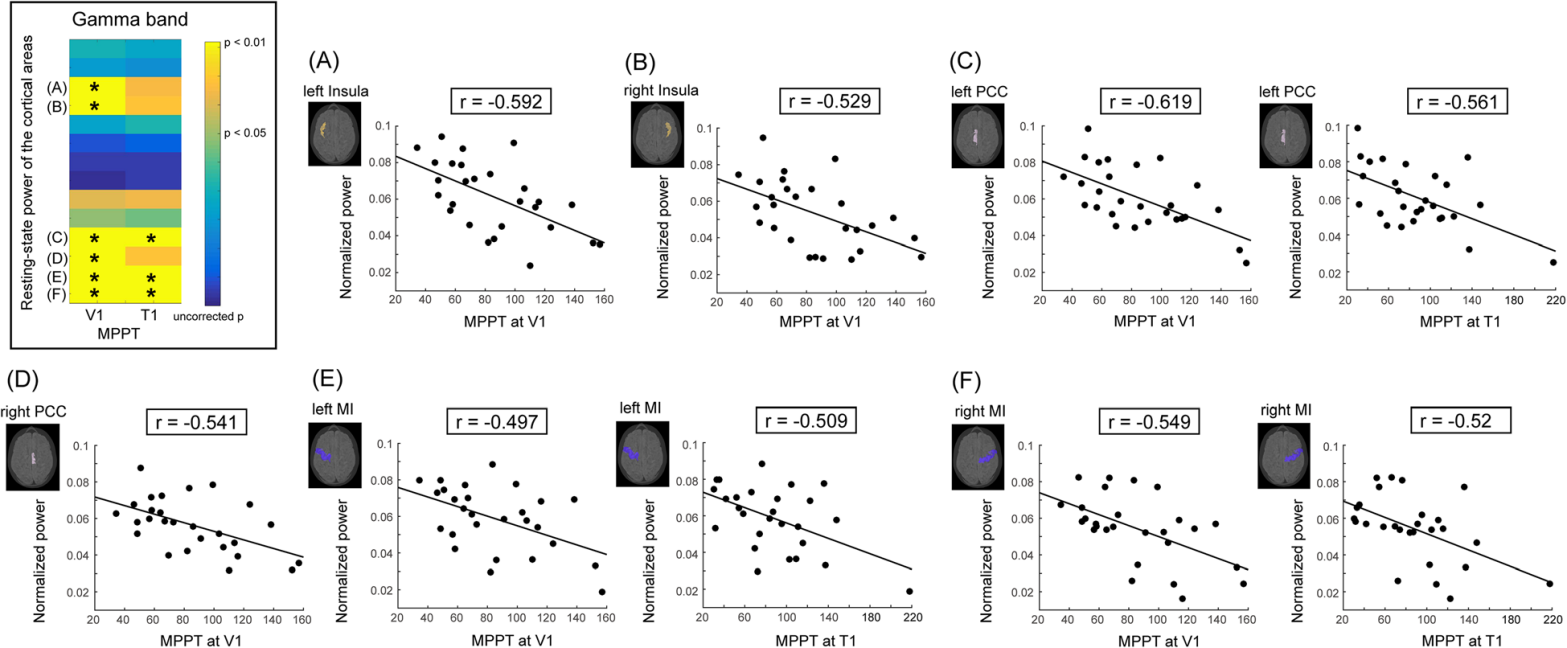
Significant correlation of the mechanical punctate pain threshold with resting-state gamma activities at the (**a**) left insula, (**b**) right insula, (**c**) left PCC, (**d**) right PCC, (**e**) left MI, and (**f**) right MI in healthy individuals. *P < .05 with false discovery rate correction. MPPT, mechanical punctate pain threshold; PCC, posterior cingulate cortex; MI, primary motor cortex; V1, left supraorbital (the first branch of the trigeminal nerve dermatome); T1, proximal medio-ventral forearm (the first thoracic nerve dermatome)

In healthy individuals, absolute cortical powers within 14 pain-related regions did not correlate with the MPPT at V1 and T1 at 5 frequency bands (all corrected *P* > 0.05; Fig. 2a). However, relative power at the gamma band was significantly linked to the MPPT (Fig. 2b), but not at other frequency bands (delta to beta bands). Notably, significant negative correlations between gamma power and the MPPT were observed between MPPTs at V1 in the bilateral insula (left, *r* = − 0.592; right, *r* = − 0.529), PCC (left, *r* = − 0.619; right, *r* = − 0.541), and MI regions (left, *r* = − 0.497; right, *r* = − 0.549) and between MPPT at T1 and power in the left PCC (*r* = − 0.561) and bilateral MI regions (left, *r* = − 0.509; right, *r* = − 0.52; Fig. 3). These relationships indicated that the high gamma power of resting-state cortical activities represented a low pain threshold, that is, high pain sensitivity. Remarkably, in the auditory cortex, no clear correlation was noted between cortical oscillations and MPPT (Supplementary Table 1).

### Correlations of the MPPT with resting-state functional connectivity

**Fig. 4:**
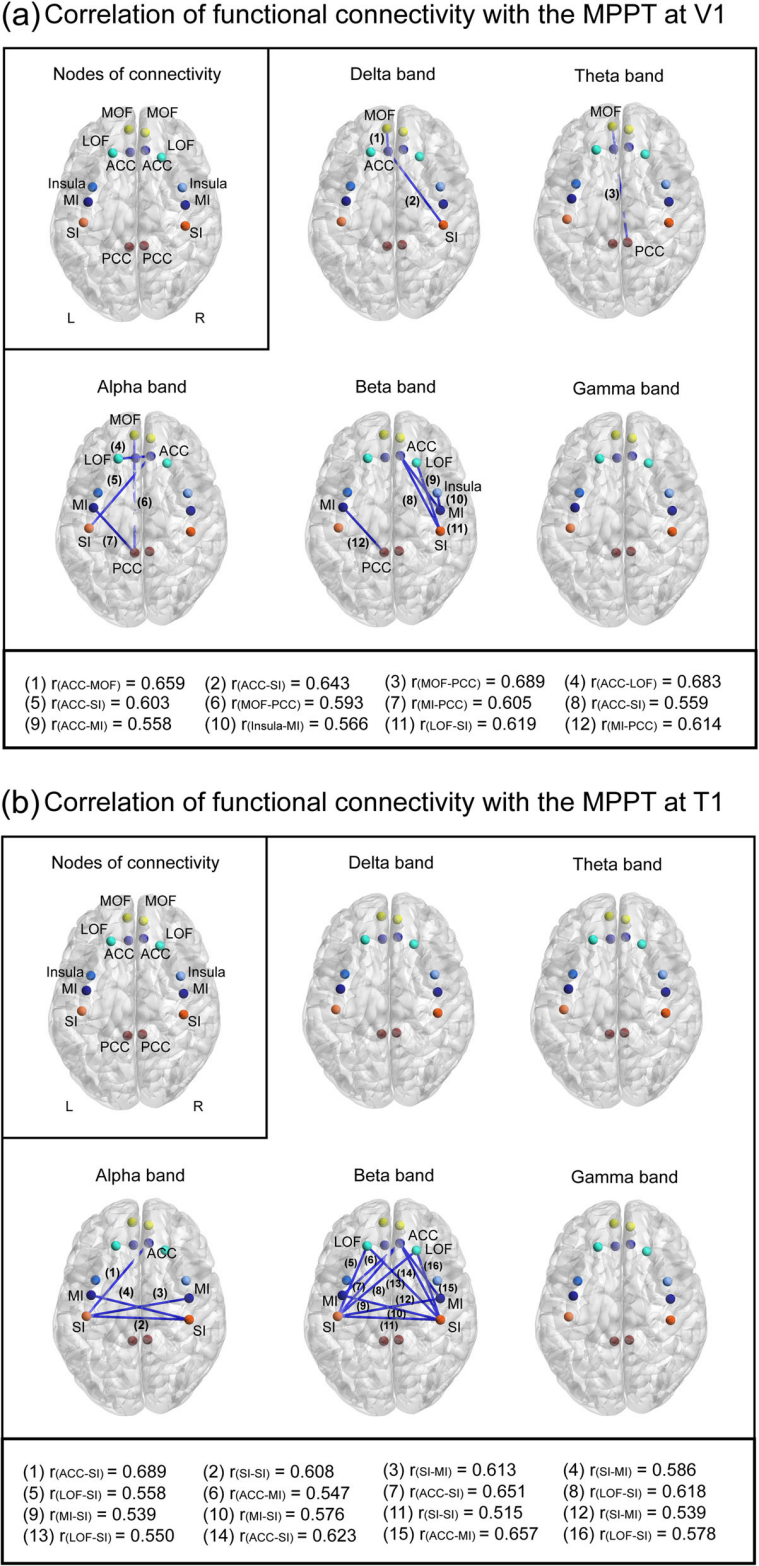
In healthy individuals, **a** The relationship between mechanical punctate pain threshold at V1 and functional connectivity within pain-related regions. Significant correlations are noted at the delta to beta bands; **b** The relationship between mechanical punctate pain threshold at T1 and functional connectivity within pain-related regions. Significant correlations are observed at the alpha and beta bands. Each node presents the centroid of each region of interest. The lower part shows the correlation coefficients of the significant connectivity. MPPT, mechanical punctate pain threshold; ACC, anterior cingulate cortex; LOF, lateral orbitofrontal cortex; MOF, medial orbitofrontal cortex; SI, primary somatosensory cortex; PCC, posterior cingulate cortex; MI, primary motor cortex; V1, left supraorbital (the first branch of the trigeminal nerve dermatome)

Significant positive correlations were observed between functional connectivity and the MPPT (*P* < 0.05 with FDR correction), indicating that strong cortical synchronization exhibited a high pain threshold. For the MPPT at V1, strong correlations were noted at the delta, theta, alpha, or beta bands within the connectivity between pain-related regions, which mainly included the cortical areas of salience network (insula and ACC), sensorimotor network (SI and MI) and some areas in the default mode network (PCC and MOF) (predominant connections are detailed in Fig. 4 (a)). Regarding the MPPT at T1, significant correlations were observed at the alpha and beta bands mainly within the connectivity at ACC and sensorimotor network (Fig. 4 (b)). Notably, the connectivity between auditory cortex at each frequency band did not correlate with the MPPT (Supplementary Table 2).

### Correlation of pain sensitivity with resting-state activities in EM

**Fig. 5:**
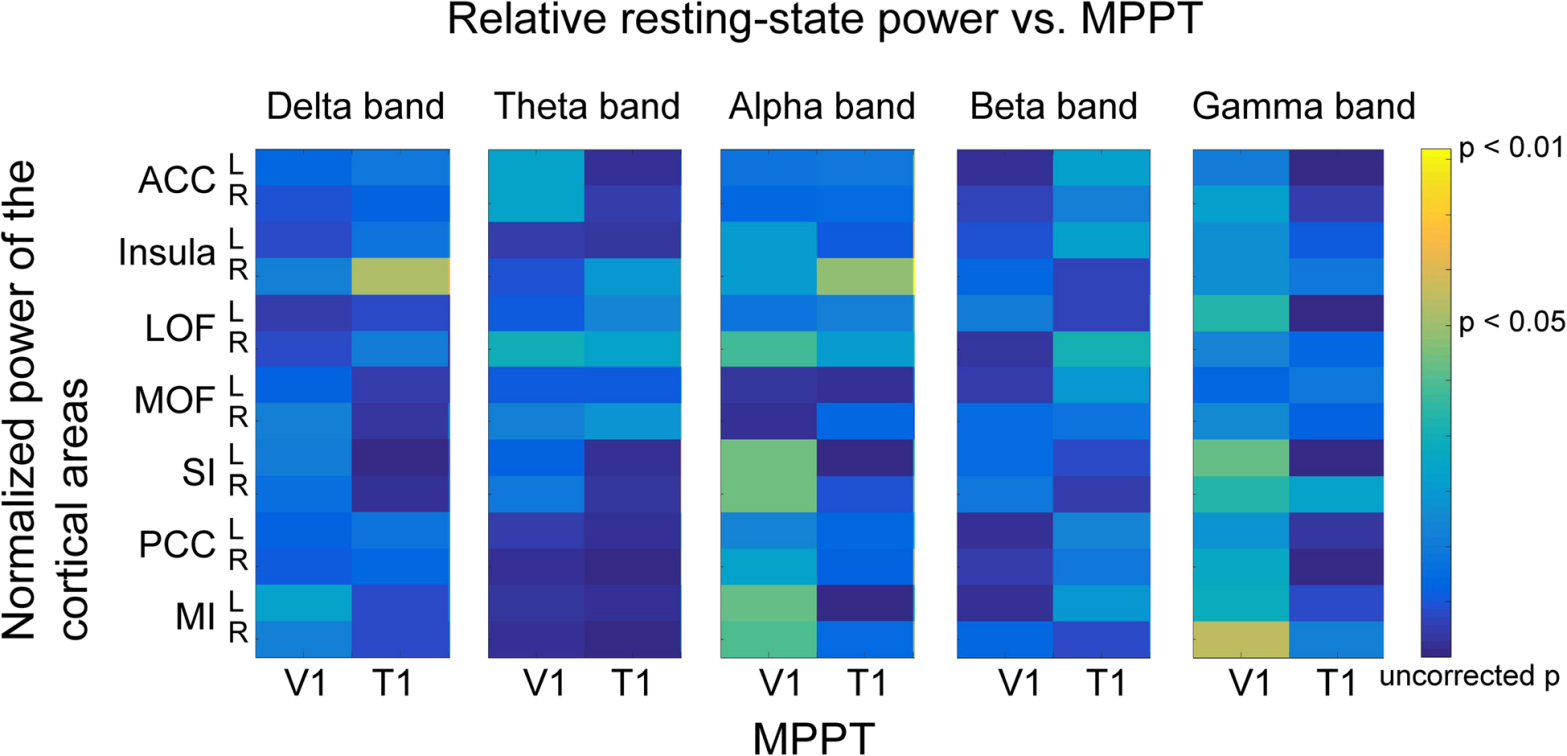
In patients with episodic migraine, no correlation between the mechanical punctate pain threshold and relative resting-state power. MPPT, mechanical punctate pain threshold; ACC, anterior cingulate cortex; LOF, lateral orbitofrontal cortex; MOF, medial orbitofrontal cortex; SI, primary somatosensory cortex; PCC, posterior cingulate cortex; MI, primary motor cortex; V1, left supraorbital (the first branch of the trigeminal nerve dermatome); T1, proximal medio-ventral forearm (the first thoracic nerve dermatome)

By contrast, in patients with EM, the power of cortical oscillations did not correlate with the MPPT at all frequency bands within the 14 ROIs (all corrected *P* > 0.05; Fig. 5). Similarly, resting-state functional connectivity between all ROIs at all frequency bands did not correlate with the MPPT (all *P* > 0.05).

## Discussion

This MEG study demonstrated that the MPPT in healthy individuals was associated with resting-state cortical oscillation and synchronization in the pain-related cortical regions, but not in the auditory cortex. Specifically, the MPPT was inversely correlated with the relative power of gamma oscillation in the bilateral insula, PCC, and MI regions and positively correlated with cortical synchronization within the pain-related cortical network. By contrast, patients with EM did not exhibit such a relationship between pain sensitivity and cortical activities.

### Association between resting-state gamma oscillation and pain sensitivity

In the present study, large gamma activities were linked to low MPPT (high pain sensitivity), implying that the resting-state gamma oscillation might be a signature of pain sensitivity. The association is in agreement with the functional roles of gamma oscillation in pain perception [45], attentional effect of pain [46], and subjective pain intensity [47]. Gamma power activities have been used to reliably encoded subjective pain ratings to ongoing experimental pain in healthy participants [48, 49] and the ongoing pain intensity in patients with chronic back pain [50]. Moreover, enhanced resting-state gamma oscillatory activity in the prefrontal cortex and cerebellum was suggested to be a characteristic marker in patients with chronic neuropathic pain [51]. In a recent animal study, gamma oscillation could increase the recruitment of neural activation in a large network between cortical and subcortical structures, and thus enhance pain perception [52]. Taken together, pain perception may be characterized by resting-state gamma oscillation, and elevated gamma activities may facilitate the neural coupling of nociceptive input.

This study further found that gamma activities underlying mechanical pain perception were observed predominantly in the bilateral insula, PCC, and MI. Moreover, a multivariate analysis of laser-evoked pain-related EEG activities suggested the objective neural correlate of individual pain sensitivity from FCz and Cz electrodes [49]. Another electrophysiological approach in humans and rodents demonstrated that the gamma event-related synchronizations sampled using Cz electrodes reliably predict pain sensitivity across individuals [48]. The findings that used EEG FCz and Cz electrodes to coded pain sensitivity [48, 49] could imply that the nociceptive-related insula and MI regions may involve the pain sensitivity mechanism. This partly agreed with the fMRI findings that indicated that insula activation coded pain intensity [53]. Additionally, regarding the difference in brain structure in the PCC, pain sensitivity was related to cortical thickness [12] and gray matter density [13]. Because of discrepancies in the recording (EEG, MRI, or fMRI vs MEG) and experimental design (task-evoked vs spontaneous activities), cortical involvement in pain sensitivity remains debatable; however, spontaneous gamma activities at the insula, MI, and PCC are suggested to be neural signatures underlying the pain sensitivity mechanism.

### Brain synchrony coupling pain sensitivity

The MPPT was coupled with cortical synchronization within the pain-related network. This echoed previous findings that prestimulus frontoparietal connectivity [21] or insular-brainstem connectivity [20] was related to subsequent pain perception. Moreover, a recent resting-state fMRI study determined that the medial-frontal and frontal-parietal networks were linked to the pain threshold [54]. Regarding the cortical function in pain stimulation, induced hemodynamic responses in the SI, ACC, and prefrontal regions reflected the subjective experience of pain [15]. Moreover, the functional connectivity in the prefrontal cortex and PCC, which are engaged in executive control and the default mode network, respectively, was associated with change in pain sensitivity for sleep disruption [55]. Similarly, the relationship between differences in brain structure and pain sensitivity was observed in the PCC, SI, and frontal regions [12, 13]. The interaction between prefrontal or ACC and sensorimotor regions has been suggested to characterize the affective or cognitive modulation of pain perception [56, 57]. Moreover, the nociceptive process was shaped from the interaction within the sensorimotor network [58, 59]. Accordingly, individual differences in pain sensitivity might result from the integration of sensory, affective, and cognitive states of the individual, indicating that oscillatory connectivity in the pain-related network could underpin pain sensitivity.

### Aberrant neural mechanism for pain sensitivity in EM

The elevated MPPT in EM echoed our recent published findings [60], suggesting the altered pain sensitivity for the neuropathology of migraine, which was further evidenced for the disassociation between the MPPT and resting-state cortical oscillations or cortical synchronizations in the present study. Notably, in consistent with our findings, Schwedt and Chong [61] examined the correlations between heat pain thresholds and cortical thickness using functional MRI, and revealed a significant negative correlation in healthy subjects but not in patients with migraine. In part, this dissociation may reflect the decreased cortical excitability [26, 27] during the interictal period and abnormal resting-state cortical oscillations and connectivity in pain-related regions [28–30] in patients with migraine. Specifically, in a resting-state condition, patients with migraine were associated with reorganized functional connectivity in the default mode, salience, or sensorimotor network [29, 62], implying that the functions of the resting brain were reshaped by clinical pain.

### Study limitations

One limitation to this study is the generalizability of the present findings to pain sensitivity with respect to other sensory modalities (eg, cold pain, hot pain, or pressure pain). Evidence suggests a close relationship between thermal and mechanical pain sensitivity [63]. However, a comparative study across sensory modalities is necessary, and this issue is beyond the scope of the present study. In addition, the processing of pain sensitivity may differ between bodily parts, as suggested by earlier psychophysical studies [64]. The varying spatial acuity of pain, innervation density, and receptive field size of nociceptors across different regions may be potential reasons for this phenomenon. Nevertheless, functional interactions within the pain-related regions considerably reflect pain sensitivity at these bodily parts.

While previous migraine studies [29, 65, 66] studying the relationship of high frequency oscillations with aberrant pain processing, the sampling rate (600 Hz) in this study limits the calculation of the high frequency oscillation (> 200 Hz). However, the power and functional connectivity at high gamma band (60–100 Hz) was further analyzed for the correlations with the MPPT. The results show no significant correlation at this frequency band (all corrected *p* > 0.05; Supplementary Tables 3–5). Furthermore, the analysis of brain activity of radial sources from cingulate cortex may be difficult with MEG. In previous MEG studies, task-related [67] and resting-state [68] ACC connectivity were clearly observed using depth-weighting MNE analysis and 306-channel whole-head Elekta Neuromag system (the same device as the present study). The reasons that we could clearly measure the MEG activity from cingulate cortex may be: (1) the source orientation in the cingulate cortex is not all radial; (2) depth-weighting algorithm alleviates the reduction of the radial source amplitude with increasing depth [69]. Herein, we delineated the spectral magnitude of the resting-state cortical activities from one healthy subject as shown in the Supplementary Fig. 1. The ACC activities are clearly discernible and the activation strengths are ranked in the middle across all cortical regions.

Finally, the dissociation between pain sensitivity and cortical activities in migraine is assumed to be due to the dysfunction of the resting-state pain network; however, the current study design was incapable of deciphering whether this dissociation in migraine is due to disease per se or its heterogeneous phenotype (headache profiles, psychiatric comorbidities, etc.).

## Conclusion

Mechanical pain sensitivity in healthy individuals is associated with the resting-state gamma oscillation and functional connectivity within pain-related brain networks. The relationship between cortical activation and pain sensitivity is not noted in EM for cortical reorganization. Resting-state cortical activities may be a brain signature of pain sensitivity in healthy individuals, and confirmatory studies in a large population are necessary.

## Supplementary Information

**Additional file 1.**

## Data Availability

The datasets analyzed during the current study are available from the corresponding author on reasonable request.

## Abbreviations

MEG: Magnetoencephalography
EM: Episodic migraine
ACC: Anterior cingulate cortex
MOF: Medial orbitofrontal
LOF: Lateral orbitofrontal
SI: Primary somatosensory cortex
MI: Primary motor cortex
PCC: Posterior cingulate cortex
MPPT: Mechanical punctate pain threshold
V1: First branch of the trigeminal nerve dermatome
T1: First thoracic nerve dermatome
MIDAS: Migraine Disability Assessment
HADS-A: The anxiety subscale of the Hospital Anxiety and Depression Scale
HADS-D: The depression subscale of the Hospital Anxiety and Depression Scale
MRI: Magnetic resonance imaging
EOG: Electrooculography
ECG: Electrocardiography
ROIs: Regions of interest
