# Supplementary material for: Individual pain sensitivity is associated with resting-state cortical activities in healthy individuals but not in patients with migraine: a magnetoencephalography study
Source: J Headache Pain. 2020 Nov 16;21(1):133. doi: 10.1186/s10194-020-01200-8 (PMC7670775; doi:10.1186/s10194-020-01200-8)
Supplement: Supplementary file 1 — Additional file 1. [file 10194_2020_1200_MOESM1_ESM.docx]

Supplementary Table 1 Relationship of the mechanical punctate pain threshold with relative

power at the delta to gamma bands in the bilateral auditory cortices in healthy individuals

|  | V1 | | | | | T1 | | | | |
| --- | --- | --- | --- | --- | --- | --- | --- | --- | --- | --- |
|  | delta | theta | alpha | beta | gamma | delta | theta | alpha | beta | gamma |
| Left  Auditory  Cortex | r = 0.012  p = 0.954 | r = -0.006  p = 0.976 | r = 0.214  p = 0.285 | r = -0.437  p = 0.023 | r = -0.356  p = 0.068 | r = 0.211  p = 0.291 | r = 0.201  p = 0.314 | r = 0.022  p = 0.915 | r = -0.402  p = 0.038 | r = -0.308  p = 0.118 |
| Right  Auditory  Cortex | r = 0.256  p = 0.197 | r = 0.156  p = 0.438 | r = -0.050  p = 0.805 | r = -0.489  p = 0.010 | r = -0.413  p = 0.032 | r = 0.341  p = 0.082 | r = 0.171  p = 0.394 | r = -0.130  p = 0.520 | r = -0.447  p = 0.019 | r = -0.318  p = 0.107 |

Note: p value is uncorrected.

V1, left supraorbital; T1, proximal medio-ventral forearm

Supplementary Table 2 Correlations between mechanical

Punctate pain threshold and functional connectivity

within bilateral auditory cortices in healthy individuals

|  | Connectivity between Bilateral Auditory Cortex | | | | |
| --- | --- | --- | --- | --- | --- |
|  | delta | theta | alpha | beta | gamma |
| V1 | r = 0.380  p = 0.050 | r = -0.047  p = 0.814 | r = -0.214  p = 0.284 | r = -0.218  p = 0.275 | r = -0.057  p = 0.778 |
| T1 | r = 0.240  p = 0.228 | r = -0.006  p = 0.975 | r = -0.245  p = 0.217 | r = -0.289  p = 0.144 | r = -0.201  p = 0.314 |

Note: p value is uncorrected.

V1, left supraorbital; T1, proximal medio-ventral forearm

Supplementary Table 3 Correlations (r value) of the high-gamma (60-100 Hz) functional connectivity with the MPPT at V1

|  |  | ACC | | Insula | | LOF | | MOF | | SI | | PCC | | MI | |
| --- | --- | --- | --- | --- | --- | --- | --- | --- | --- | --- | --- | --- | --- | --- | --- |
|  |  | L | R | L | R | L | R | L | R | L | R | L | R | L | R |
| ACC | L | NaN | -0.138 | 0.304 | 0.218 | 0.313 | 0.267 | 0.230 | 0.261 | -0.133 | 0.323 | -0.175 | 0.024 | -0.128 | 0.265 |
|  | R | -0.138 | NaN | 0.032 | -0.065 | 0.271 | 0.098 | 0.032 | -0.001 | -0.133 | 0.268 | -0.151 | -0.138 | -0.094 | 0.135 |
| Insula | L | 0.304 | 0.032 | NaN | 0.150 | 0.378 | 0.089 | 0.007 | 0.005 | -0.188 | 0.193 | 0.160 | 0.246 | -0.068 | 0.194 |
|  | R | 0.218 | -0.065 | 0.150 | NaN | 0.275 | 0.141 | 0.050 | 0.005 | -0.029 | 0.337 | 0.118 | 0.241 | -0.002 | 0.199 |
| LOF | L | 0.313 | 0.271 | 0.378 | 0.275 | NaN | 0.258 | 0.161 | 0.098 | 0.130 | 0.259 | 0.255 | 0.280 | 0.006 | 0.248 |
|  | R | 0.267 | 0.098 | 0.089 | 0.141 | 0.258 | NaN | 0.106 | 0.164 | 0.024 | 0.400 | 0.159 | 0.240 | -0.168 | 0.281 |
| MOF | L | 0.230 | 0.032 | 0.007 | 0.050 | 0.161 | 0.106 | NaN | -0.398 | 0.011 | 0.187 | 0.188 | 0.257 | -0.133 | 0.157 |
|  | R | 0.261 | -0.001 | 0.005 | 0.005 | 0.098 | 0.164 | -0.398 | NaN | -0.114 | 0.173 | 0.147 | 0.299 | -0.152 | 0.149 |
| SI | L | -0.133 | -0.133 | -0.188 | -0.029 | 0.130 | 0.024 | 0.011 | -0.114 | NaN | -0.003 | 0.139 | 0.103 | 0.031 | -0.064 |
|  | R | 0.323 | 0.268 | 0.193 | 0.337 | 0.259 | 0.400 | 0.187 | 0.173 | -0.003 | NaN | 0.252 | 0.318 | 0.006 | 0.096 |
| PCC | L | -0.175 | -0.151 | 0.160 | 0.118 | 0.255 | 0.159 | 0.188 | 0.147 | 0.139 | 0.252 | NaN | -0.222 | -0.027 | 0.186 |
|  | R | 0.024 | -0.138 | 0.246 | 0.241 | 0.280 | 0.240 | 0.257 | 0.299 | 0.103 | 0.318 | -0.222 | NaN | -0.178 | 0.286 |
| MI | L | -0.128 | -0.094 | -0.068 | -0.002 | 0.006 | -0.168 | -0.133 | -0.152 | 0.031 | 0.006 | -0.027 | -0.178 | NaN | -0.067 |
|  | R | 0.265 | 0.135 | 0.194 | 0.199 | 0.248 | 0.281 | 0.157 | 0.149 | -0.064 | 0.096 | 0.186 | 0.286 | -0.067 | NaN |

Supplementary Table 4 Correlations (r value) of the high-gamma (60-100 Hz) functional connectivity with the MPPT at T1

|  |  | ACC | | Insula | | LOF | | MOF | | SI | | PCC | | MI | |
| --- | --- | --- | --- | --- | --- | --- | --- | --- | --- | --- | --- | --- | --- | --- | --- |
|  |  | L | R | L | R | L | R | L | R | L | R | L | R | L | R |
| ACC | L | NaN | -0.182 | -0.045 | -0.102 | 0.002 | -0.041 | -0.099 | -0.056 | -0.192 | 0.018 | -0.289 | -0.100 | -0.199 | -0.045 |
|  | R | -0.182 | NaN | -0.189 | -0.247 | -0.010 | -0.179 | -0.190 | -0.218 | -0.235 | 0.007 | -0.310 | -0.205 | -0.228 | -0.103 |
| Insula | L | -0.045 | -0.189 | NaN | -0.137 | 0.108 | -0.158 | -0.191 | -0.181 | -0.227 | -0.053 | -0.097 | -0.079 | -0.204 | -0.102 |
|  | R | -0.102 | -0.247 | -0.137 | NaN | -0.015 | -0.122 | -0.189 | -0.146 | -0.188 | 0.083 | -0.170 | -0.035 | -0.156 | -0.078 |
| LOF | L | 0.002 | -0.010 | 0.108 | -0.015 | NaN | -0.076 | -0.051 | -0.052 | -0.043 | -0.004 | -0.040 | -0.019 | -0.162 | -0.016 |
|  | R | -0.041 | -0.179 | -0.158 | -0.122 | -0.076 | NaN | -0.160 | -0.140 | -0.179 | 0.043 | -0.106 | -0.074 | -0.246 | -0.018 |
| MOF | L | -0.099 | -0.190 | -0.191 | -0.189 | -0.051 | -0.160 | NaN | -0.405 | -0.166 | -0.042 | -0.110 | -0.056 | -0.247 | -0.112 |
|  | R | -0.056 | -0.218 | -0.181 | -0.146 | -0.052 | -0.140 | -0.405 | NaN | -0.250 | -0.040 | -0.137 | -0.035 | -0.253 | -0.085 |
| SI | L | -0.192 | -0.235 | -0.227 | -0.188 | -0.043 | -0.179 | -0.166 | -0.250 | NaN | -0.136 | 0.023 | -0.141 | -0.001 | -0.197 |
|  | R | 0.018 | 0.007 | -0.053 | 0.083 | -0.004 | 0.043 | -0.042 | -0.040 | -0.136 | NaN | -0.049 | 0.034 | -0.198 | -0.180 |
| PCC | L | -0.289 | -0.310 | -0.097 | -0.170 | -0.040 | -0.106 | -0.110 | -0.137 | 0.023 | -0.049 | NaN | -0.247 | -0.135 | -0.096 |
|  | R | -0.100 | -0.205 | -0.079 | -0.035 | -0.019 | -0.074 | -0.056 | -0.035 | -0.141 | 0.034 | -0.247 | NaN | -0.259 | -0.004 |
| MI | L | -0.199 | -0.228 | -0.204 | -0.156 | -0.162 | -0.246 | -0.247 | -0.253 | -0.001 | -0.198 | -0.135 | -0.259 | NaN | -0.170 |
|  | R | -0.045 | -0.103 | -0.102 | -0.078 | -0.016 | -0.018 | -0.112 | -0.085 | -0.197 | -0.180 | -0.096 | -0.004 | -0.170 | NaN |

Supplementary Table 5 Correlations (r value) of the high-gamma (60-100 Hz) power with the MPPT at V1 and T1

|  | ACC | | Insula | | LOF | | MOF | | SI | | PCC | | MI | |
| --- | --- | --- | --- | --- | --- | --- | --- | --- | --- | --- | --- | --- | --- | --- |
|  | L | R | L | R | L | R | L | R | L | R | L | R | L | R |
| V1 | -0.028 | 0.017 | -0.448 | -0.347 | -0.032 | 0.084 | 0.182 | 0.080 | -0.407 | -0.167 | -0.342 | -0.273 | -0.468 | -0.167 |
| T1 | -0.189 | -0.151 | -0.398 | -0.402 | -0.246 | -0.117 | -0.046 | -0.040 | -0.438 | -0.297 | -0.448 | -0.339 | -0.486 | -0.364 |

V1, left supraorbital; T1, proximal medio-ventral forearm


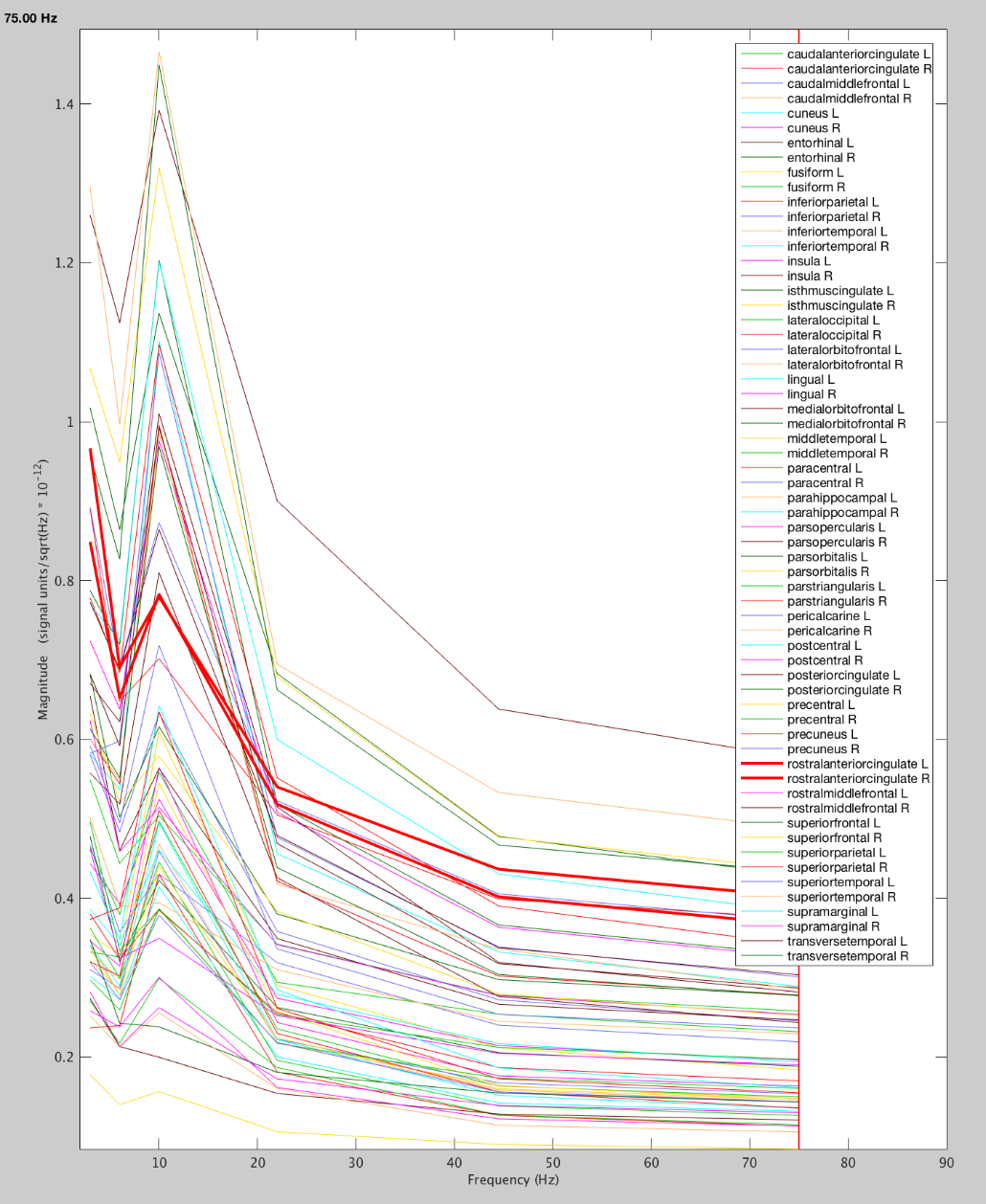


Supplementary Figure 1. The spectral magnitude of the resting-state cortical activities from one healthy subject. The red tracings that indicated bilateral ACC activities are clearly discernible and the activation strengths are ranked in the middle across all cortical regions.
